# B. Dysenteriae Shiga in Severe Cases

**Published:** 1918-05

**Authors:** 


***B. Dysenteriae Shiga in Severe Cases.*** By Dr. Fernand Bezançon, in collaboration with MM. Ranque, Senez, Coville et Paraf. Abs. from *Bulletin de l'Académie de Médecine*, tome LXXIX, No. 12.

The author reports that during a small epidemic of bacillary dysentery in a certain military sector all the serious cases were due to B. dysenteriae Shiga, while, on the contrary, in all the mild cases this organism was conspicuous by its absence. In no instance was the bacillus of Flexner nor the bacillus of Hiss isolated. Careful study of the agents responsible for the less serious cases divided them into two groups of unidentified dysenteric bacilli.

